# Differences in Semantic Memory Encoding Strategies in Young, Healthy Old and MCI Patients

**DOI:** 10.3389/fnagi.2019.00306

**Published:** 2019-11-12

**Authors:** Gil Suzin, Ramit Ravona-Springer, Elissa L. Ash, Eddy J. Davelaar, Marius Usher

**Affiliations:** ^1^The School of Psychological Sciences, Tel Aviv University, Tel Aviv, Israel; ^2^The Sagol Center for Hyperbaric Medicine and Research, Assaf Harofeh Medical Center, Zerifin, Israel; ^3^Sackler Faculty of Medicine, Tel Aviv University, Tel Aviv, Israel; ^4^Memory and Psychogeriatric Clinics, Sheba Medical Center, Ramat-Gan, Israel; ^5^Department of Neurology, Tel Aviv Sourasky Medical Center, Tel Aviv, Israel; ^6^Department of Psychological Sciences, Birkbeck, University of London, London, United Kingdom

**Keywords:** aging, MCI, associative memory, eye tracker, adjusted ratio of clustering

## Abstract

Associative processes, such as the encoding of associations between words in a list, can enhance episodic memory performance and are thought to deteriorate with age. Here, we examine the nature of age-related deficits in the *encoding of associations*, by using a free recall paradigm with visual arrays of objects. Fifty-five participants (26 young students; 20 cognitive healthy older adults; nine patients with Mild Cognitive Impairment, MCI) were shown multiple slides (experimental trials), each containing an array of nine common objects for recall. Most of the arrays contained three objects from three semantic categories, each. In the remaining arrays, the nine objects were unrelated. Eye fixations were also monitored during the viewing of the arrays, in a subset of the participants. While for young participants the immediate recall was higher for the semantically related arrays, this effect was diminished in healthy elderly and totally absent in MCI patients. Furthermore, only in the young group did the sequence of eye fixations show a semantic scanning pattern during encoding, even when the related objects were non- adjacent in the array. Healthy elderly and MCI patients were not influenced by the semantic relatedness of items during the array encoding, to the same extent as young subjects, as observed by a lack of (or reduced) semantic scanning. The results support a version of the *encoding of the association* aging-deficit hypothesis.

## Introduction

While it is well-established that episodic memory declines with age, this decline is not uniform, and it affects some tasks and processes more than others. For example, the largest age-related memory declines are found in tasks that depend on retrieval strategies, such as free-recall; in comparison, smaller deficits are found in recognition memory (Rabinowitz, 1984; Craik and McDowd, 1987). Although this disparity suggests a retrieval deficit, there is also indication of an encoding deficit, as older participants are less likely to form rich, elaborative memory traces (Rabinowitz and Ackerman, 1982; Craik et al., 1987; Craik and Jennings, 1992).

Aging deficits in episodic memory have often been explained by frontal mechanisms (Moscovitch and Winocur, 1995; West, 1996; Head et al., 2009). As reviewed in detail by West (1996), this hypotheses is supported by behavioral studies showing significant correlations between neuropsychological measures of frontal lobe functions and memory tests sensitive to aging (Parkin and Walter, 1992; Troyer et al., 1994; Grieve et al., 2007), as well as by neurobiological evidence showing an age-related differential reduction in neural processes within the pre-frontal cortex (PFC; Haug and Eggers, 1991; Gunning-Dixon et al., 2008). As the PFC is thought to play a role in memory control (Moscovitch and Winocur, 1995; Shimamura, 1995; Elhalal et al., 2014), a frontal mechanism hypothesis predicts that age-induced deficits in memory would resemble the deficits of patients with frontal lobe dysfunction. Indeed, similarly to patients with frontal lobe dysfunction, older adults show deficits in tests of free-recall, but not in memory recognition (Janowsky et al., 1989; Gershberg and Shimamura, 1995; Baldo and Shimamura, 1998; Luo and Craik, 2008). In particular, older adults, like frontal patients show a deficit in the retrieval of temporal information (Shimamura et al., 1990; Kahana et al., 2002; Golomb et al., 2008), and in clustering semantically related words in free recall (Delis et al., 1987; Düzel et al., 2010; Cadar et al., 2018). A frontal deficit in aging is also consistent with the *associative memory* deficit hypothesis (Naveh-Benjamin, 2000; Naveh-Benjamin et al., 2007), which has been used to explain age deficits in memory recognition of word-pair lists. Accordingly, item association is a basic mechanism of encoding, which weakens with age.

Here, we examine the role of semantic associations in memory, using a novel experimental paradigm of free recall with visual arrays (instead of word lists), which allows us to monitor the spontaneous encoding strategies of the memory material. Instead of word-lists, we used visual arrays of objects that are simultaneously presented and we vary their semantic relatedness (see also Moar, 1977). By using a visual array instead of standard word sequences, we did not impose a temporal structure on the memory material and thus, the participants had full control over the encoding strategies (spatial or semantic) of the information. This gave us a unique opportunity to probe the encoding strategies used spontaneously by participants. Previous research on visual search from arrays of objects has shown that pre-existing associations between objects promote the deployment of attention, thereby facilitating their recognition compared with non-related objects (Moores et al., 2003) and helping to determine the location within the display to look at next (Henderson and Hollingworth, 1999).

In particular, we wanted to test how age affects the ability to spontaneously encode semantically related visual arrays. The memory literature shows conflicting results on the impact of knowledge (e.g., semantic structure of material) on aging deficits. While studies that probed memory on pair-associates reported that semantic structure reduces aging deficits (Naveh-Benjamin et al., 2003; see review in Umanath and Marsh, 2014), especially when knowledge provides specific cues at retrieval (Badham et al., 2016), free-recall studies have reported an opposite effect: younger subjects benefit more from semantic structure than older ones (Heron and Craik, 1964; Craik and Masani, 1967; Cadar et al., 2018). Moreover, semantic clustering in a free-recall is lower in older adults compared with younger controls (Norman et al., 2000; Barker-Collo et al., 2002; Stricker et al., 2002; Taconnat et al., 2009; Cadar et al., 2018). Based on this, we expected that aging would reduce the ability of the participants to spontaneously deploy semantic clustering strategies in our free-recall visual array task, resulting in reduced semantic enhancement effects. There are two ways in which such strategies may be implemented. First, participants may (consciously) self-initiate a semantic encoding strategy, and second, they may (without awareness), automatically deploy attention driven by associative links between objects. We will return to this distinction in the “Discussion” section. To track the encoding process, we monitored the eye-scanning trajectory in a subset of participants as they viewed the display. We expected that younger subjects will progressively scan the memory arrays based on semantic relations, while older subjects will keep relying on spatial scanning. Finally, to understand how this deficit depends on the severity of the memory degradation, we also tested, in addition to young and healthy older participants, a group of patients with Amnestic Mild Cognitive Impairment (aMCI), a condition known to precede Alzheimer’s disease (AD). We expected that due to their marked cognitive impairments (i.e., memory performance 1.5 SD below corresponding age and education norms; Carlesimo et al., 1998; Morris and Price, 2001; Holland et al., 2009), aMCI patients would display an even lower semantic enhancement effect in comparison to older adults without cognitive impairment.

## Materials and Methods

### Participants

Three groups of participants were included in the current research.

#### Younger Group

This included 26 psychology students participating for credit or payment (14 women; mean age = 25, range = 18–31; mean education = 16). Students were included in the sample if they were under 35 years old with reported normal or corrected-to-normal vision, had no background of health conditions and were native Hebrew speakers.

#### Healthy Older Group

The group included 20 healthy older participants, recruited from retirement homes or senior citizen centers, either voluntarily or for payment (13 women; mean age = 72, range = 65–86; mean education = 15). According to the inclusion criteria, participants were selected if they were above 60 years of age with normal or corrected-to-normal vision, and native Hebrew speakers. All participants had to pass a visual acuity test, and score 28 and above on the Mini-Mental State Examination (MMSE). This conservative cut-off score was chosen to ensure optimal rates of specificity (O’Bryant et al., 2008), as lower scores may indicate undiagnosed MCI. The operational definition of normal cognition was established after the presence of any neurological conditions was ruled out and it was established that the participant was not being treated with prescribed anti-dementia medicine. Finally, participants were only included in the sample if they did not suffer from acute unstable health conditions (participants were interviewed and screened for neurological, cardiovascular, diabetic and psychiatric conditions).

#### aMCI Group

This group included nine patients (two women; mean age = 77, range = 66–84; mean education = 14), diagnosed with AMCI, a condition well known to precede Dementia. Patients were recruited from memory clinics in two major hospitals in the Tel Aviv area after being referred by physicians (psychiatrists or neurologists) specializing in diagnosis and treatment of dementia and related conditions. aMCI was diagnosed based on the accepted Petersen criteria (Petersen et al., 1999). All aMCI patients were above 60 years old with normal or corrected-to-normal vision, and were native Hebrew speakers. All participants passed a visual acuity test. They were only included in the sample if they did not suffer from acute unstable health conditions (participants were interviewed and screened for neurological, cardiovascular, diabetic and psychiatric conditions). They were also included only if they scored 27 or below on MMSE, indicating almost certain cognitive impairment (O’Bryant et al., 2008). All participants were examined by an independent psychiatrist who confirmed their ability to understand the instructions of the experiment.

None of the participants, regardless of group, were aware of the purpose of the experiment. The study protocol was approved by the Tel Aviv University Institutional Review Board, the Sheba Medical Center Review Board and the Tel-Aviv Sourasky Medical Center Institutional Review Board. All participants signed a written informed consent. Trials were conducted at Tel Aviv University and at the Goldschleger Eye Institute at the Sheba Medical Center.

### Stimuli

**Figure 1:**
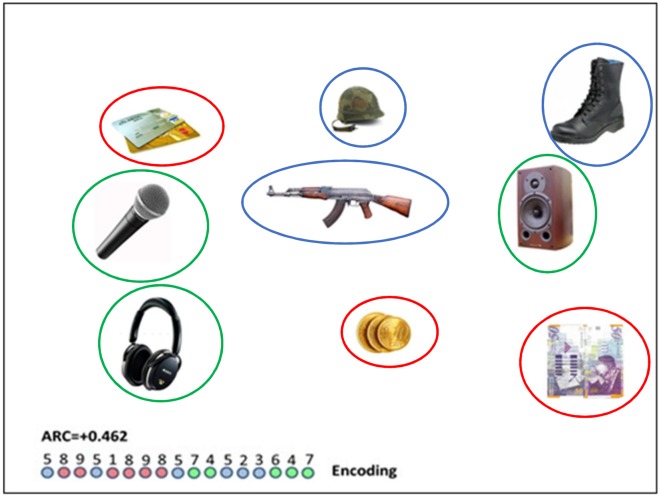
A semantically related slide (i.e., red circles indicate “payment methods”; blue circles indicate “military equipment” and green circles show “audio devices”); colored circles appear in this photo for illustration purposes and did not appear in the experiment. Number sequence relates to the trajectory of eye gaze moving between numbered objects. The encoded clusters involves the repeated colors (8–9);(1–8–9–8);(7–4);(5–2–3);(6–4–7). For this particular sequence, the encoding clustering index (ARC; see “The Eye-Tracking” section below) is 0.462. The (longer) diameter of the objects, as they appeared on the screen was in the range of 100–160 pixels, and the distance between the center of objects was in the range of 350–450 pixels.

Participants were shown multiple slides, each containing nine common objects. Some of the slides consisted of objects that were selected from three semantically-related categories (e.g., music-audio devices, money, military equipment; see Figure 1) with three objects from each category. In other slides, the objects were selected from different/unrelated categories^1^. In all the slides, objects were randomly spatially arranged. In order to show that the semantic-related slides and the non-related slides differed in perceived relatedness, two judges who were unaware of the objective of the experiment were requested to rate relatedness of sets of three objects; half of the sets corresponded to the object categories and half of them contained objects from different and unrelated categories. As they were presented with these sets of three objects, the judges were asked to evaluate their “relatedness” on a scale of 1–10. The relatedness of semantic sets was higher than of non-related sets (8.8 vs. 1.8, *p* < 0.001). The interrater reliability was high (*r* = 0.83).

Stimuli were generated using Matlab and were presented on a gamma-corrected ViewSonic (Walnut, CA, USA) 17-inch. CRT monitor, viewed at a distance of 100 cm. The screen resolution was set to 1,024 × 768 pixels, and the monitor had a refresh rate of 60 Hz.

### Procedure

Each participant was shown three blocks of 20 slides. Each slide contained nine common objects. In 16 of the 20 slides in each block, objects were arranged in broadly semantically related groups (three semantic groups for a total of nine objects; see Figure 1 for an example). In the rest of the slides, objects were arranged in semantically unrelated groups. Each group was repeated in the 2nd and 3rd blocks in a new (randomized) spatial organization and with a new combination of other groups (for example, if in block one, slide-1 contains, groups, 1, 2, 3, and slide-2, groups, 4, 5, 6, in block-2, slide 1 may contain group 1, 4, 7, etc…). Each trial began with a fixation cross displayed for 1.5 s; after that, a slide containing objects was presented for 7 s. Participants were requested to memorize the objects in order to verbally free recall as many of them as they could. Free recall began immediately after the presentation of objects ended. Recall data was collected by the experimenter before continuing to the next trial. There was no time limit set for recall of objects and the progression to the next trial was manually controlled by the experimenter when the participant indicated to have no more items to report^2^.

For 10 younger participants, 20 healthy, older participants and seven MCI patients, the experiment procedure was identical and supplemented with monitoring of eye fixation using an eye-tracker device. Eye movements were recorded with an EyeLink 1000 infrared system (SR Research, Kanata, ON, Canada). The sampling rate was set to 500 Hz with a spatial resolution of less than 0.01°. Recording was from the right eye only, though viewing was binocular. Participants sat 1 m in front of a computer screen, while their head was placed in a head-stabilizing device. The camera was placed underneath the computer screen, unnoticeable to the participants who were watching the slides. Before each block, the eye tracker device was calibrated using a standard 9-point calibration routine (Bonneh et al., 2015). Following calibration, the participants were asked to move only their eyes, and not their heads, during the experiment. A 2-min rest was given before re-calibration and progression to the next block. For six participants (two young, four healthy elderly), there was an equipment malfunction. Therefore, the eye-tracking results are based on the data from eight young, 16 older adults, and seven participants with MCI.

#### Eye-Tracking Analysis: ARC

For eight younger participants, 16 healthy older participants and 7 MCI patients a sequence of eye fixations on objects was obtained for each slide. In order to do so, the objects slides were superimposed on a location grid with fixed locations, whereas, the upper row represents locations “1”-“2”-“3” (from left to right), the middle row represents locations “4”-“5”-“6” (from left to right) and the bottom row represents locations “7”-“8”-“9” (from left to right). The criteria used for detecting fixations include: (1) a gaze that lasts 100 ms. or longer, that is located on a region of interest (ROI), which is centered on each object with a radius of 100 pixels. These characteristics are commonly used in research measuring fixation points with an eye-tracker device (i.e., Raney et al., 2014), and the ROI was big enough to include the objects (longer diameter of objects in the range of 100–160 pixels) and to separate them from each other. One such example of an eye-scan sequence is illustrated in Figure 1. In the semantic clusters slide trials, the sequences were used to compute an encoding semantic clustering measure using the Adjusted Ratio of Clustering measure (ARC) score (Roenker et al., 1971), which is a clustering measure based upon recall-based expectancy, i.e., it computes a “normalized” deviation from chance by conditioning on the number of items reported from each category. The ARC-score is computed as:

ARC = (OBS−EXP)/(MAX−EXP)

where: *OBS*: is the number of cluster pair repetitions in a trial (in the Figure 1 illustrated trial there are nine cluster pair repetitions corresponding to the repeated colors in the encoding circle-sequence: 8–9; 1–8; 8–9; 9–8; 7–4; 5–2; 2–3; 6–4; 4–7), *MAX* is the maximum number of cluster pair repetitions, had all the fixations of one category been organized together before moving on to fixating on items from the next category. Max is calculated by subtracting the number of categories participating in a trial (three categories in our example) from the total number of fixations (17 − 3 = 14). *EXP* is the number of category repetitions expected by chance, conditioned, on the number of items, *n*_i_, scanned from each category, and on the sequence length, *r*.

$EXP=\sum_{i=1}^{3}\frac{\left[n_{i}(n_{i}-1)\right]}{r}$ where *n*_i_ = the number of fixations attended from each category. In this trial, EXP = [(6*5) + (6*5) + (5*4)]/17 = 80/17 = 4.71. Note that the denominator in the EXP formula is needed for normalization. Thus, for the trial, illustrated in Figure 1, EXP = [(6*5) + (6*5) + (5*4)]/17 = 80/17 = 4.71, and the normalized score is ARC = (9–4.71)/(14–4.71) = 0.462.

We also computed for the younger group, a number of additional clustering measures in order to contrast between spatial and semantic clustering strategies. In particular, we computed a row-based (spatial) ARC score (by considering the three rows instead of the three semantic categories, as clusters) and a semantic-index that excludes adjacent transitions. As there is no closed-form formula for the EXP-value of this semantic index when adjacent transitions are excluded, we used for this purpose an input type semantic measure (see Stricker et al., 2002), which allows a simple estimation of the number of semantically clustered objects expected by chance. In each trial, we count the number of transitions between semantically related objects in the sequence after excluding all adjacent transitions (and repetitions) and we divide by the number of nonadjacent transitions and subtract the chance level (2/8). The semantic/non-adjacent index for the sequence in Figure 1 would yield a score of 2/5 − 2/8 = 0.15 (two nonadjacent, but semantic, transitions out of five non-adjacent transitions). Finally, we have computed a relative “semantic minus location” index, in which we subtracted for each trial, the number of transitions that are adjacent and non-semantic from the number of transitions that are semantic and non-adjacent (and divided by the total number of transitions in the trial). As this index is a relative one, and we use it to probe changes in encoding strategies across blocks, we do not compute a chance level for it.

## Results

Fifty-five participants completed the behavioral procedure (26 younger, 20 healthy older, nine MCI). The groups did not differ in years of education, and the two older groups did not differ in age (see Table 1). The healthy older group received higher MMSE scores on average. The MCI group was characterized by a smaller female/male ratio (more males than other groups; see Table 1 for group differences).

**Table 1:** Group differences-mean (Standard Deviation).

	Young (*N* = 26)	Healthy elderly (*N* = 20)	aMCI (*N* = 9)	Significance
Age (years)	25.4 (3.56)	72.36 (5.74)	76.75 (6.05)	N.S (*)
Education (years)	15.75 (2.25)	14.83 (2.29)	14.25 (4.77)	N.S
MMSE	-	29.45 (0.88)	25.11 (1.36)	*P* < 0.001 (*)
Sex (female/male ratio)	1.16	2	0.222	-

(*) Comparison was made only between the two older groups.

**Figure 2:**
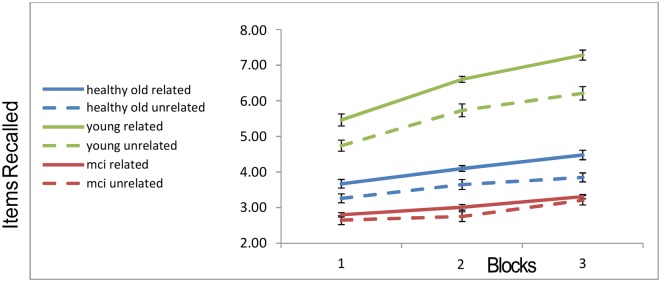
Recall rates of the three groups across blocks. “Error-bars” correspond to within-participants standard-errors.

The memory recall as a function of the group, block and semantic condition (related vs. unrelated) are shown in Figure 2.

A 3 × 3 × 2 mixed ANOVA^3^ with within-subject factors block and trial type (related vs. unrelated) and a between-subject factor of group, revealed a main effect of block (with more objects recalled as blocks progressed; *F*_(2,104)_ = 118.668, *p* < 0.001), a main effect of trial-type (more objects recalled from related slides compared to unrelated slides; *F*_(1,52)_ = 58.986, *p* < 0.001), a main effect of group (*F*_(2,52)_ = 97.618, *p* < 0.001). Younger participants recalled more objects than the healthy older participants, who in turn recalled more than the MCI patients. In addition, the interaction of group and block was significant (*F*_(4,104)_ = 21.586, *p* < 0.001), as well as the interaction of group and relatedness (*F*_(2,52)_ = 9.718, *p* < 0.001). In order to ensure that these interactions are not completely attributable to the MCI group, we ran a further 2 (young, healthy-old) × 2 (related, unrelated) ANOVA. Both the interaction between semantic relatedness and age-group, and that between block and group, remain highly significant, even when the MCI patients are excluded (interaction of group and block (*F*_(2,88)_ = 28.7, *p* < 0.001); interaction of group and semantic-relatedness (*F*_(1,44)_ = 8.93, *p* < 0.006). The recall-improvement (increase in recall from block 1 to 3) was larger for the younger group compared to the healthy older (*t*_(44)_ = 7.935, *p* < 0.001) and MCI groups (*t*_(33)_ = 6.87, *p* < 0.001). There was no difference in recall-improvement between the two older groups (*t*_(27)_ = 1.29, N.S.; see Figure 2). Finally, for the young group, the recall was higher in the semantically related condition as compared to the unrelated condition (*t*_(25)_ = 9.99, *p* < 0.001). Healthy older adults still showed a relatedness advantage (*t*_(19)_ = 5.16; *p* < 0.001), but it was not as large as that of the younger participants. By contrast, the MCI participants showed no relatedness advantage (*t*_(8)_ = 1.014, *p* = 0.34; see Figure 2). This effect is unlikely to be caused by a “floor-effect,” as trials in which MCI participants recalled no objects were less than 4%.

### Encoding Strategies

Eight young adults, 16 healthy elderly and seven MCI patients completed the behavioral procedure while their eye gaze was monitored. For each participant, an eye movement semantic clustering score (ARC) was calculated on each trial to detect whether visual encoding (traced *via* eye movements) followed a semantic scanning sequence. Statistical analysis was done on the semantic related clusters trials of the 2nd and 3rd blocks, as we considered the first block a necessary practice to allow the participants to establish their search strategy. Indeed, there was no difference (*F*_(2,32)_ = 0.126, *p* = 0.882) between the three groups in the ARC of the first block (which was negative), indicating an initial array scanning strategy based on spatial rather than semantic structure^4^.

**Figure 3:**
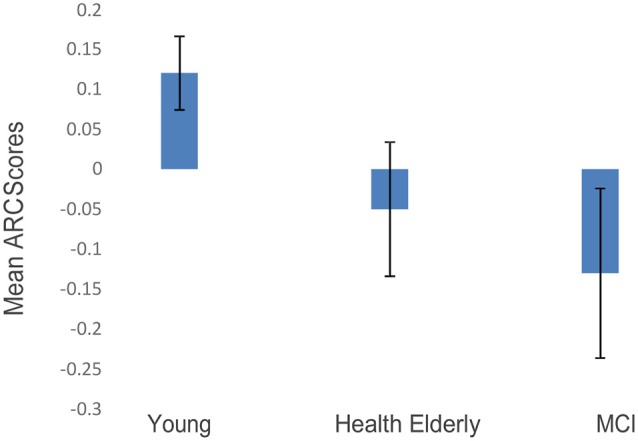
ARC scores of the three age groups (blocks 2 + 3 collapsed; error bars correspond to standard errors of the mean).

The post-practice ARC scores in blocks 2 + 3 were entered in a one-way ANOVA model, with the group as a between-subjects’ variable, comparing the three groups’ ARC-means on the two final blocks (Blocks 2 and 3 collapsed). A significant effect of group was found (*F*_(2,27)_ = 9.673, *p* < 0.002; see Figure 3). Fisher’s *post hoc* comparisons revealed a significant difference between ARC scores of the younger group compared to the healthy older group (Mean = 0.11, SD = 0.06 vs. Mean = −0.04, SD = 0.1, *p* < 0.008), and also a significant difference between the younger group and the MCI group (Mean = 0.11, SD = 0.06 vs. Mean = −0.12, SD = 0.14, *p* < 0.001). The MCI’s ARC scores were numerically lower than those of the healthy elderly, but no significant difference was found between the two older groups. Importantly, only the younger group showed significantly positive ARC scores, indicating that the scanning strategy of the younger participants was guided by a semantic structure.

**Figure 4:**
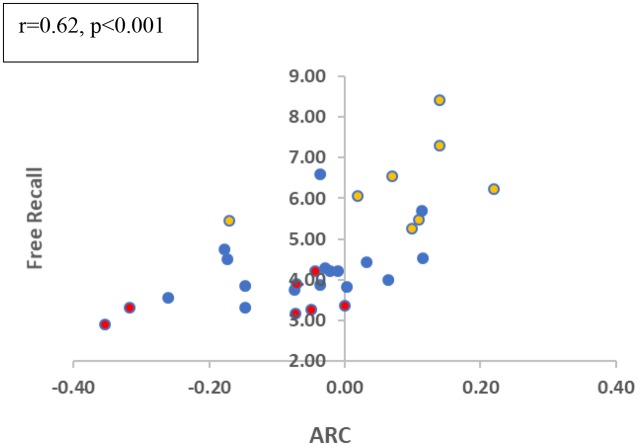
Free recall in related trials and ARC correlation (blocks 2 + 3 collapsed). Colors refer to the different groups (Yellow-Young subjects/Blue-Healthy Elderly/Red-MCI).

Finally, we examined the correlation between ARC-scores and total-recall across the participants (we again focused on block 2–3 collapsed, as block 1 seems to be required to gain task proficiency). As shown in Figure 4, the two measures show a moderately-high correlation (*r* = 0.62; *p* < 0.001). Participants who deploy a semantically related scanning pattern show a higher recall performance. This correlation is significant (*r* = 0.50, *p* = 0.013) when the MCI group is eliminated, and it remains marginally significant when it includes only the two elderly groups (*r* = 0.45; *p* = 0.06), which show (at the group level) ARC-scores close to zero. This suggests that small variations in clustering at encoding affect memory performance even in these subjects^5^. Finally, we carried out a regression analysis for the recall in the related conditions (blocks 2 and 3), based on two predictors, the ARC-score and the age of the participant. The results show that the two predictors explained 69.8% of the variance (*R*^2^ = 0.698, *F*_(2,25)_ = 28.953, *p* < 0.001). Moreover, both predictors are significant: Age (*β* = −0.636, *p* < 0.001) and ARC (*β* = 0.301, *p* < 0.029 for ARC), indicating that semantic encoding contributes to recall on top of just age.

Finally, in order to better understand how the semantic strategy develops across practice with the task, we examined in blocks 2 and 3 (which have positive semantic-ARC for the young group) two indexes that reflect spatial and semantic strategies of scanning the array. The first index is a “Semantic NonAdjacent” (SNA) index, which estimates a (corrected to chance) measure of transitions that are semantic and nonadjacent. The second is a “Semantic minus Adjacent” (S−A) index, which subtracts the number of adjacent non-semantic transitions from the number of semantic non-adjacent ones (and divides by the number of transitions in each trial). Note that since there are more possible adjacent transitions than semantic one, this index is expected to be negative, however, increments (across blocks) corresponds to shift in strategy from spatial to semantic.

As we predicted, apriori tests show an increase from block-2 to block-3 for the young participants on both indexes (SNA: *t*_(7)_ = −2.68, *p* < 0.05; S−A: *t*_(7)_ = −4.73, *p* < 0.005), suggesting that the young participants transit from a spatial to a semantic scanning strategy. For the healthy-old participants, the SNA score is at chance and does not change with block, while the S−A score shows only a marginal change in the healthy old group (S−A: *t*_(17)_ = −1.99, *p* = 0.063), and no change at all in the MCI patients (S−A: *t*_(6)_ = −0.85, *p* = 0.935). The overall interaction, however, was not significant (*p* = 0.169).

## Discussion

We examined the memory performance of three groups of participants (young, healthy older adults, and patients with MCI) on a free-recall task with visual arrays of objects that were either structured (three groups of related objects) or not (nine unrelated objects). Since we use a free-recall with supra-span arrays (without a delay or distractor interval between the stimuli presentation and the recall), the recall involves both WM and LTM components (Waugh and Norman, 1965; Davelaar et al., 2005). We did not aim to separate these components here, but we examined how aging affects memory recall, with particular emphasis on encoding processes. Younger participants showed higher recall rates than healthy older participants, and the older participants showed higher recall than MCI patients. The repetition of object-sets benefited recall in the younger group more than in the two older groups, as indicated by a block × group interaction. This phenomenon was observed for unrelated objects also, suggesting (as we argue below) that healthy aging, and all the more so aging accompanied by MCI deficits is characterized by both encoding and retrieval memory deficits. While retrieval deficits can also account for a reduced recall score with age, the interaction with block suggests that young participants are able to better encode the objects, when they see them again in blocks 2 and 3. This is because the retrieval requirements across the three blocks are the same, whereas the encoding is enhanced (by the mere repetition and, possibly, by the opportunity the subjects have to trigger idiosyncratic associations between nominally unrelated items; Tulving, 1964). According to this logic, when we see the same object again we may encode it better, but its retrieval process remains the same (one retrieves the object, based on the same display). However, it is possible to argue that seeing the same object again, results also in retrieval changes (say, due to an increase in interference). While we admit the possibility of such retrieval changes, we believe they are minor compared with encoding advantages caused by seeing the same material again. Thus, in order to more fully support a specific encoding deficit, we now discuss the interaction of age with semantic relations and the encoding strategies as measured by eye-tracking.

**Figure 5:**
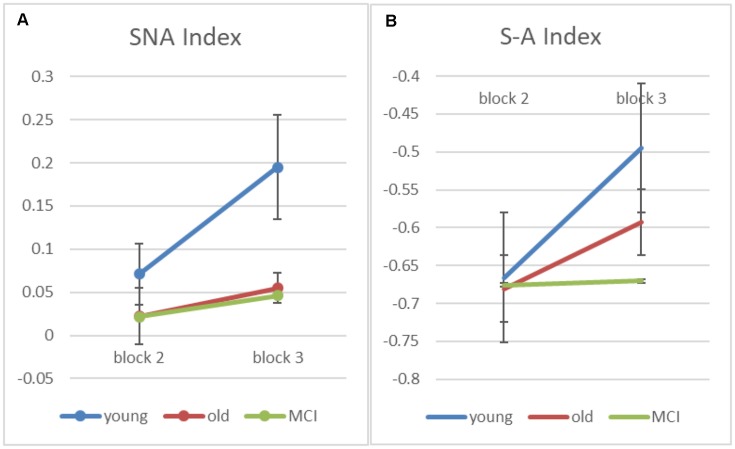
**(A)** Input-based clustering score counting only semantic non-adjacent (SNA) transitions for blocks 2 and 3 (error bars correspond to standard errors of the mean). **(B)** A computed score—“Semantic minus Adjacent” (S−A) index, which subtracts adjacent non-semantic transitions from the number of semantic non-adjacent ones (and divides by the number of transitions in each trial).

The memory enhancement that participants obtained from spontaneously encoding and retrieving the objects based on semantic relations was of particular interest in our study. Since we did not explicitly probe first-person reports from participants, saying whether or not they intentionally used a semantic strategy to encode the objects, we cannot dissociate here between a self-initiated intentional encoding and an automatic deployment of attention driven by associative links between objects. Based on informal debriefing and on the fact that automatic deployment of attention to pre-existing associations was demonstrated even in visual search tasks (where the associations do not promote performance; Moores et al., 2003), we tend to attribute the semantically clustering effects to the latter. However, we believe that both factors may contribute and future research (with a larger number of participants) will be needed to evaluate their relative contribution. What we found is that semantic relations between the visual objects enhanced the recall rates for the younger group and (to a lesser degree) for the healthy older group, but not for the MCI patients. Furthermore, eye movement trajectories during the viewing of the arrays suggested different encoding strategies. All participants in block-1 showed negative semantic ARC-values, which indicate a default spatial scanning strategy. In later blocks, however, while younger participants appeared to view the memory arrays based on a semantic scanning strategy, older participants from both groups used such a strategy to a lesser extent. Indeed, as shown in Figures 3, 5, the younger participants show a shift from a spatial to a semantically scanning strategy. These results are consistent with an age-related encoding deficit that prevents older participants from attending to the semantic relations among the objects (as indicated by the ARC scores of the eye trajectories in Figure 3), resulting in a reduced relatedness effect in free recall (Figure 2). This interpretation is supported by the correlation between semantic ARC-scores, and total-recall in the related trials.

We need to interpret this correlation result with caution, as a correlation does not demonstrate causality. It is possible, for example, that aging independently (i.e., by different mechanisms) affects both memory recall and semantic encoding. There are few reasons, however to believe the semantic encoding and memory recall are causally related. First, as we reported above, the semantic encoding (as measured by the encoding-ARC) predicts recall in the related condition, even after aging is factored out. Second, we find that the encoding-ARC is also correlated with the improvement in memory (δ-Sem) as a result of related items [Recall (related) – Recall (unrelated); *r* = 0.36, *p* = 0.04; 1-tailed, without the MCI group, and *r* = 0.275, *p* = 0.07; 1-tailed, for all subjects]. As δ-Sem already subtracts general memory differences, this correlation is more specific to the potential semantic encoding deficits^6^.

Despite these results, a more careful examination of the data, suggests a more complex picture. As one can see in Figure 2, the semantic relatedness improved memory recall already in the first block. However, there were no between-group differences in the ARC of the first block, and this score was negative, indicating that the visual scanning pattern was not influenced by semantic relations between objects. This suggests that the effect of semantic relatedness on memory recall is not (only) the result of using a semantic scanning pattern, at least (not) in the first block. It is possible that the participants showed an increased effect of semantic relatedness in blocks 2 and 3 because they were more able to deploy automatic attention resources to semantic/associative links between objects, which may have been learned in previous blocks. Reactivated associations between objects may have then influenced the visual scanning pattern during encoding. To test this *post hoc* explanation we correlated the δ-Sem in block-1 with the ARC in blocks 2 and 3. The results show a marginal correlation for block-2 (*r* = 0.30, *p* < 0.10, 2-tailed) and a significant correlations for block-3 (*r* = 0.36; *p* < 0.048, 2-tailed). While these results are tentative, they suggest a more nuanced picture of a potential two-way interaction between memory recall and the deployment of semantic strategies, which should be examined in future studies.

Turning to the age differences, the healthy older participants’ smaller semantic effect (solid vs. dashed blue lines in Figure 2), may thus reflect, either the partial encoding of semantic relations between adjacent objects in the array, or alternatively could reflect a retrieval benefit (i.e., one object helping to retrieve another *via* semantic priming), which is less affected by age (Laver and Burke, 1993; Mehta and Jerger, 2014). Finally, the total lack of a relatedness effect and the lower baseline recall in the unrelated condition in the MCI group (solid and dashed red lines in Figure 2) is consistent with a stronger memory deficit that affects the encoding of both individual items and their inter-relations, as well as the retrieval processes. Previous studies found such severe deficits to be associated with atrophy in frontal and hippocampal sites (Haug and Eggers, 1991; Stebbins et al., 2002; Grady et al., 2003; Apostolova et al., 2006).

The results of our experiment are consistent with those of previous free-recall studies (Heron and Craik, 1964; Craik and Masani, 1967; Cadar et al., 2018) which reported increased aging deficits for semantically related items. This effect is opposite to the decreased deficit that was reported with pair-associates tasks. We believe that the difference stems from the fact that free-recall requires the deployment of spontaneous encoding strategies (what item to encode with what), whereas in pair-associate memory older adults can benefit from instructions to rely on prior knowledge (Umanath and Marsh, 2014, p.416–7). The same aging deficit is also observed in the reduced semantic clustering in the CVLT task (Delis et al., 1987; Cadar et al., 2018; CVLT manual). The results are also consistent with a version of the encoding of association aging deficit hypothesis (i.e., Naveh-Benjamin, 2000; Naveh-Benjamin et al., 2007; Wegesin et al., 2000), which points to the encoding of associations between items as the most critical process that is affected by aging. While the encoding of association deficit is usually applied to the encoding of unrelated items into episodic memory, here we suggest that it also applies to the encoding of weakly associated items, in tasks that require a spontaneous strategy for organizing the material.

To conclude, using a novel memory paradigm which allows the participants full control over their encoding strategies, we demonstrated that aging affects the ability to attend and encode the semantic relations among objects in a memory set. This deficit is enhanced in MCI patients.

## Ethics Statement

The study protocol was approved by the Tel Aviv University Institutional Review Board, the Sheba Medical Center Review Board and the Tel-Aviv Sourasky Medical Center Institutional Review Board.

## Author Contributions

GS, MU and ED conceived the study idea. GS and MU designed the experiment. RR-S and EA supervised aMCI patients recruitment. GS collected the data. GS and ED analyzed the data. GS and MU wrote the manuscript. All of the authors substantially helped in revising the manuscript and preparing it for publishing. All of the authors approved the manuscript before submission.

## Conflict of Interest

The authors declare that the research was conducted in the absence of any commercial or financial relationships that could be construed as a potential conflict of interest.

## Footnotes

^1^All the objects were high-familiarity objects (*N* =180, in total), selected from Google-images. Out of these objects, there was a group of 144 objects, which could be grouped into semantic categories of three objects each (3×48 = 144 related objects). The other 36 objects were unrelated (see test below). There was no difference in the word-familiarity (estimated *via* the MRC psycholinguistic database) between the related and unrelated objects: Mean (SD) Related = 553 (50); Unrelated = 558 (37); *p* = >0.5.

^2^We were careful to allow all participants, in particular the elderly ones, as much time as they needed for the recall.

^3^All tests are 2-tailed, unless specifically reported otherwise.

^4^A row-based ARC score in the first block showed a positive value of 12 (SE = 0.08) for the eight young participants. These scores were numerically positive for six out of the eight. In contrast to the negative semantic ARC, this indicates an initial bias for a spatial scanning strategy.

^5^Due to the small number of young participants that carried out the eyetracking procedure we cannot assess the statistical significance of this correlation for the young group alone (yellow circles in Figure 4).

^6^Note that since we only had four unrelated trials per block (as opposed to 16 related trials), the delta measure is noisier than the related recall measure, accounting for one source for the reduction in correlation.
